# Elevated Soluble VEGF Receptor sFlt-1 Correlates with Endothelial Injury in IgA Nephropathy

**DOI:** 10.1371/journal.pone.0101779

**Published:** 2014-07-09

**Authors:** Ya-Ling Zhai, Li Zhu, Su-Fang Shi, Li-Jun Liu, Ji-Cheng Lv, Hong Zhang

**Affiliations:** 1 Renal Division, Department of Medicine, Peking University First Hospital, Beijing, China; 2 Peking University Institute of Nephrology, Beijing, China; 3 Key Laboratory of Renal Disease, Ministry of Health of China, Beijing, China; 4 Key Laboratory of Chronic Kidney Disease Prevention and Treatment (Peking University), Ministry of Education, Beijing, China; Fondazione IRCCS Ospedale Maggiore Policlinico & Fondazione D′Amico per la Ricerca sulle Malattie Renali, Italy

## Abstract

**Background:**

Endothelial injury, which may present clinically as hypertension, proteinuria and increased von Willebrand Factor (vWF) level, is a common manifestation in IgA nephropathy (IgAN). However, causal factors for endothelial injury in IgAN are not completely understood. An imbalance of vascular endothelial growth factor/Soluble fms-like tyrosine kinase-1 (VEGF/sFlt-1) has been observed in many diseases with endothelial dysfunction, including pre-eclampsia and diabetic retinopathy, but whether it contributes to endothelial injury in IgAN requires further exploration.

**Methods:**

Initially, 96 IgAN patients and 22 healthy volunteers were enrolled as a discovery cohort. VEGF/sFlt-1, sFlt-1 and VEGF levels were compared between patients with IgAN and healthy volunteers to explore the underlying factors that contribute to endothelial injury in IgAN. The identified contributor (sFlt-1) was further confirmed in a replication cohort, which included 109 IgAN patients and 30 healthy volunteers. Correlations of sFlt-1 with hypertension, proteinuria, Oxford-E score and plasma vWF were further evaluated in the combined 205 patients with IgAN.

**Results:**

VEGF/sFlt-1 levels were significantly lower in IgAN patients than healthy volunteers (0.33±0.27 *vs.* 0.43±0.22, p = 0.02) in the discovery cohort. Within the ratio, plasma sFlt-1 levels were significantly elevated (101.18±25.19 *vs.* 79.73±18.85 pg/ml, p<0.001), but plasma VEGF levels showed no significant differences. Elevated sFlt-1 levels in the replication cohort were confirmed in IgAN patients (93.40±39.78 *vs.* 71.92±15.78 pg/ml, p<0.001). Plasma sFlt-1 levels in IgAN patients correlated with proteinuria (severe (>3.5 g/d) *vs.* moderate (1–3.5 g/d) *vs.* mild (<1 g/d) proteinuria: 115.95±39.09 *vs.* 99.89±28.55 *vs.* 83.24±33.92 pg/ml; severe *vs.* mild: p<0.001, moderate *vs.* mild p = 0.001, severe *vs.* moderate: p = 0.014), hypertension (with *vs.* without hypertension: 107.87±31.94 *vs.* 87.32±32.76 pg/ml, p = 0.015) and vWF levels (r = 0.161, p = 0.021).

**Conclusions:**

The present study found elevated sFlt-1 in IgAN patients and further identified its correlation with proteinuria, hypertension and vWF levels. These results suggested that elevated sFlt-1 contributes to endothelial injury in IgAN.

## Background

IgA nephropathy (IgAN) is the most common primary glomerulonephritis worldwide[1]. The peak age of the clinical onset of IgAN is during the second and third decade of life[2]. Approximately half of the IgAN patients present with hypertension or a history of hypertension at disease onset despite their young age[3], and some patients present with secondary malignant hypertension[4]. Recently, Karoui et al. reported frequent (up to 53%) thrombotic microangiopathy (TMA) lesions in IgAN patients that may occur in normotensive patients with near-normal renal histology, which excluded severe hypertension or advanced renal disease as sole causes and left the underlying pathophysiological mechanisms undetermined[5]. Moreover, many studies show that plasma von Willebrand Factor (vWF), a specific marker for endothelial cells injury, is abnormal in patients with IgAN, who exhibit elevated levels or defective molecules[6], [7]. The high prevalence of hypertension, frequently observed TMA and elevated circulating vWF indicate vascular endothelial injury in IgAN. However, the underlying mechanism has not been discovered.

Soluble fms-like tyrosine kinase-1 (sFlt-1 or sVEGFR-1) is a soluble splice variant of the vascular endothelial growth factor (VEGF) receptor, which is produced by several tissues. sFlt-1 lacks a transmembrane and cytoplasmic domain, and it negatively regulates the actions of VEGF, a highly specific mitogen for vascular endothelial cells, by binding free circulating VEGF and occupying the VEGF receptor. VEGF and sFlt-1 constitute a balance of angiogenic and anti-angiogenic factors in human circulation[8]–[10]. An imbalance between an angiogenic agent (VEGF) and an anti-angiogenic factor (sFlt-1), as reflected by VEGF/sFlt-1, has been reported in many diseases[11]–[13]. Pre-eclampsia is the most popular condition. The elevated sFlt-1 in pre-eclampsia patients leads to widespread endothelial dysfunction and clinical findings of proteinuria and hypertension, which are often observed in many glomerular diseases. Angiogenic imbalance was also reported in diabetic retinopathy patients, and this imbalance was further associated with the fate of diabetic macular edema[14]. Kim et al. found that the urinary excretion of sFlt-1 increased at a relatively early stage in diabetic nephropathy, and it was associated with urinary albumin excretion[12]. Recently, Di et al. reported that excess sFlt-1 was associated with endothelial dysfunction in CKD patients, which implies the involvement of an angiogenic imbalance in kidney disease[15].

Few studies have explored the mechanism of vascular endothelial injury in IgA nephropathy. Our previous studies found that the prevalence of anti-endothelial cell antibodies (AECA) was higher in IgAN patients with severe intra-renal arterial lesions and malignant hypertension[16], [17]. AECA cannot explain all of the endothelial dysfunction in patients with IgAN. Therefore, we speculated that the presence of other risk factors, such as an angiogenic imbalance of VEGF/sFlt-1, may be involved in these processes. The present study investigated whether patients with IgAN exhibited imbalanced VEGF and sFlt-1 expression and whether this imbalance contributed to endothelial injury in IgAN.

## Subjects and Methods

### Study population

Two independent cohorts, a discovery cohort and replication cohort, were included in the present study (Figure S1). In the discovery cohort, 96 plasma-available IgAN patients (diagnosed in 2006–2007) and 22 healthy volunteers with normal urine analysis and blood pressure were enrolled. The replication cohort included 109 plasma-available IgAN patients (diagnosed in 2011–2012) and 30 healthy volunteers.

The granular deposition of IgA in the glomerular mesangium using immunofluorescence detection and the deposition of electron-dense material in the mesangium using ultra-structural examination confirmed the diagnosis of IgAN. Detailed clinical and laboratory examinations excluded patients with Henoch-Schonlein purpura, systemic lupus erythematosus, and chronic hepatic diseases.

Plasma (EDTA anticoagulated) was collected, on the morning that IgAN patients received a renal biopsy or the healthy volunteers were included in the study. Plasma samples were divided into aliquots and stored at −80°C for measurements of sFlt-1, VEGF and vWF levels. Blood pressure and 24-hour urine protein excretion at the time of renal biopsy were collected from medical records. Hypertension was defined as the average of two blood pressure readings on a single occasion with a systolic blood pressure >140 mmHg or diastolic blood pressure >90 mmHg or patients who reported use of antihypertensive medication. The estimated glomerular filtration rate (eGFR) was evaluated using the Modified Glomerular Filtration Rate Estimating Equation for Chinese[18]. Percutaneous renal biopsy was performed in every IgAN patient. The severity of renal lesions of patients with IgAN was graded according to the Oxford classifications[19], [20]. Biopsy adequacy was defined as a minimum of 8 glomeruli available for light microscopy examination. Two renal pathologists blinded to the clinical data scored the renal histopathology from all patients using 4 pathological variables: mesangial hypercellularity (M), segmental glomerulosclerosis (S), endocapillary hypercellularity (E) and tubular atrophy/interstitial fibrosis (T).

The study protocol was approved by the Medical Ethics Committee of Peking University First Hospital and informed written consent was obtained from every participant.

### Detection of plasma sFlt-1, VEGF and vWF

The plasma levels of sFlt-1 and VEGF were detected using commercial ELISA kits according to the manufacturer's specifications (R&D Systems, Minneapolis, MN, USA). Plasma levels of vWF were determined using ELISA as previously described[16]. Briefly, ELISA plates were coated with rabbit anti-human vWF polyclonal antibodies (DAKO, Denmark) at 4°C overnight. After blocking, diluted (1∶50) test plasma was added, and vWF was detected with horseradish peroxidase (HRP)-conjugated rabbit anti-human vWF polyclonal antibodies (DAKO, Denmark). Individual vWF levels were determined from OD450 values within the linear range of the standard curve via comparison to values obtained from dilutions of purified vWF (Calbionchem, Germany)

### Statistical Analysis

Descriptive statistical analyses were performed with SPSS10.0 software (SPSS Inc., USA). Continuous variables were compared by the unpaired Student's t test or ANOVA (ANalysis Of VAriance between groups). Dichotomous and polychromous data were analysed by the χ2 test. The Spearman correlation was used to analyze correlation. Results were expressed as means ± SD or median (IQR). A p value of less than 0.05 was considered statistically significant.

## Results

### Clinical and pathological manifestations of IgAN patients

The present study enrolled a total of 205 IgAN patients, including 96 patients in the discovery cohort and 106 patients in the replication cohort. We confirmed that none of these patients received anticoagulant therapy because low molecular weight heparin administration increases circulating sFlt-1 levels[21]. The clinical and pathological manifestations were comparable between patients in the discovery and replication cohorts. The primary clinical and pathological manifestations of patients are summarized in Table 1. Within the 205 IgAN patients, 132 patients were male and 73 patients were female. The average age at renal biopsy was 32.77±10.09 years old, and 47.32% (97/205) of patients presented with hypertension. The proteinuria levels were 1.40 (0.84, 2.76) g/d. The eGFR levels of patients in this group were 82.15±38.65 ml/min/1.73 m^2^. According to the Oxford classification, 83.41% (171/205) of the patients were grade M1, 20.98% (43/205) were grade E1, 44.39% (91/205) were grade S1, 36.10% (74/205) were grade T1, and 18.05% (37/205) were grade T2.

**Table 1:** Clinical and pathological manifestations of enrolled IgAN patients.

Characteristics	Mean ± SD (IQR or percentage)		
	Discovery cohort	Replication cohort	combined cohort
Age (year)	32.94±9.96	32.62±10.25	32.77±10.09
Gender (male)	65/96 (67.71%)	67/109 (61.47%)	132/205 (64.39%)
Hypertension (%)			
with hypertension	45/96 (46.87%)	52/109 (47.71%)	97/205 (47.32%)
without hypertension	51/96 (53.13%)	57/109 (52.29%)	108/205 (52.68%)
Initial proteinuria (g/day, median, IQR)	1.59 (0.93, 3.22)	1.26 (0.79,2.47)	1.40 (0.84,2.76)
<1 (%)	26/96 (27.08%)	42/109 (38.53%)	70/205 (34.15%)
1–3.5 (%)	48/96 (50%)	54/109 (49.54%)	102/205 (49.76%)
≥3.5 (%)	22/96 (22.92%)	13/109 (11.93%)	33/205 (16.09%)
eGFR (ml/min per 1.73 m^2^)	77.27±37.26	86.44±39.58	82.15±38.65
Oxford classification (%)			
M score			
M0	16/96 (16.67%)	18/109 (16.51%)	34/205 (16.59%)
M1	80/96 (83.33%)	91/109 (83.49%)	171/205 (83.41%)
E score			
E0	72/96 (75%)	90/109 (82.57%)	162/205 (79.02%)
E1	24/96 (25%)	19/109 (17.43%)	43/205 (20.98%)
S score			
S0	62/96 (64.58%)	52/109 (47.71%)	114/205 (55.61%)
S1	34/96 (35.42%)	57/109 (52.29%)	91/205 (44.39%)
T score			
T0	63/96 (65.63%)	31/109 (28.44%)	94/205 (45.85%)
T1	15/96 (15.62%)	59/109 (54.13%)	74/205 (36.10%)
T2	18/96 (18.75%)	19/109 (17.43%)	37/205 (18.05%)

### Identification of elevated sFlt-1 in IgAN patients in the discovery cohort

Gender distribution and age were not significantly different between the enrolled IgAN patients and healthy volunteers in the discovery cohort. VEGF/sFlt-1 levels (ratio) were significantly lower in IgAN patients than healthy volunteers (IgAN *vs.* healthy volunteers: 0.33±0.27 *vs.* 0.43±0.22, p = 0.02), which indicated an imbalance of pro-angiogenesis and anti-angiogenesis. The VEGF levels were comparable in the two groups (IgAN *vs.* healthy volunteers: 31±27.12 *vs.* 31.86±13.92 pg/ml, p = 0.213), but plasma sFlt-1 levels were significantly elevated in IgAN patients (IgAN *vs.* healthy volunteers: 101.18±25.19 *vs.* 79.73±18.85 pg/ml, p<0.001), which indicated that elevated sFlt-1 contributed to endothelial injury in IgAN (Table 2).

**Table 2:** Plasma sFlt-1, VEGF, VEGF/sFlt-1 levels of enrolled IgAN patients and healthy volunteers in the discovery cohort.

	Healthy volunteers	IgAN patients	p-value
Number	22	96	
Age (y)	32.64±6.69	32.94±9.96	0.864
Gender (M/F)	14/8	65/31	0.714
VEGF/sFlt-1	0.43±0.22	0.33±0.27	0.02
sFlt-1 (pg/ml)	79.73±18.85	101.18±25.19	<0.001
VEGF (pg/ml)	31.86±13.92	31.31±27.12	0.213

### Validation of elevated sFlt-1 in IgAN patients in the replication cohort

**Figure 1:**
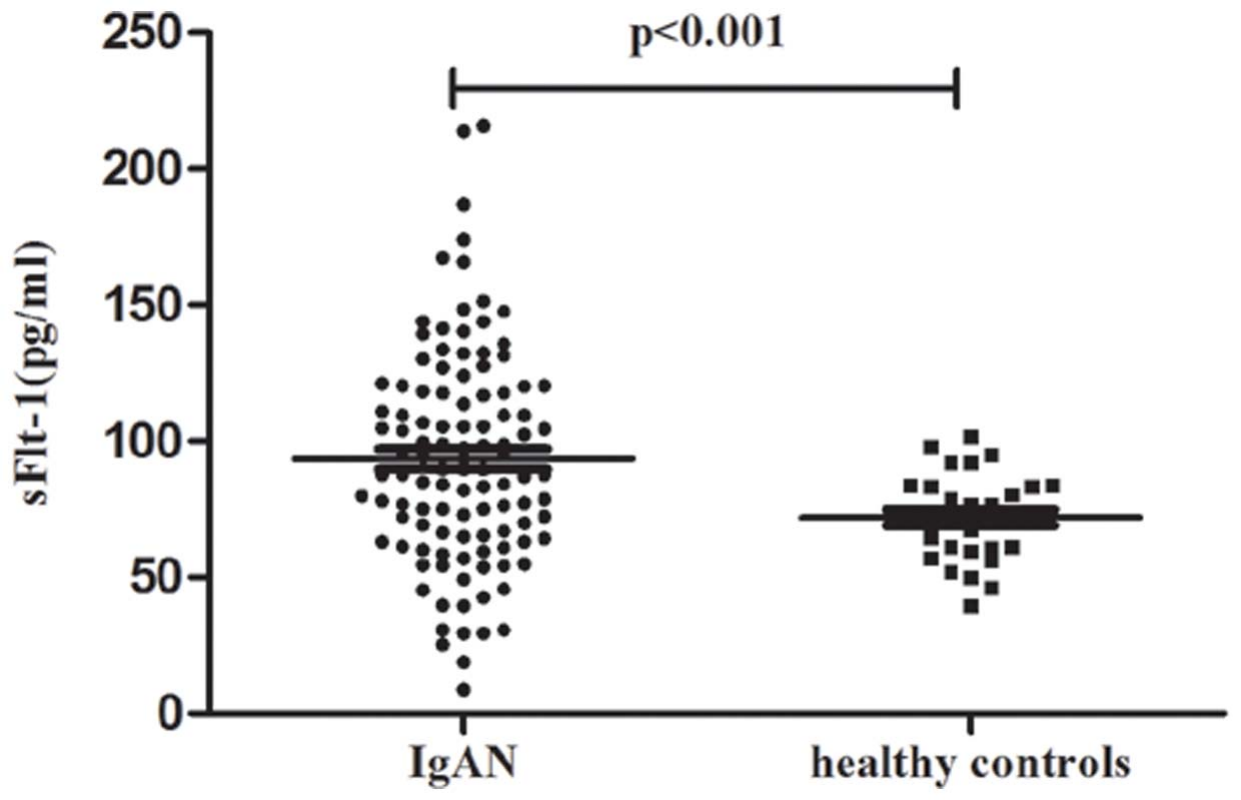
Validation of sFlit-1 levels in the replication cohort. Scatter plot showing the distribution of sFlt-1 levels in IgAN patients and healthy volunteers in the replication cohort. Patients with IgAN showed significantly elevated sFlt-1 levels compared to healthy volunteers.

We enrolled another independent cohort (the replication cohort) to confirm our identification of elevated sFlt-1 in IgAN patients. sFlt-1 levels in IgAN patients in the replication cohort were significantly higher than healthy volunteers (93.40±39.78 *vs.* 71.92±15.78 pg/ml, p<0.001; Figure 1), which was similar to the discovery cohort.

### Correlations between elevated sFlt-1 and endothelial injury in patients with IgAN

In pre-eclampsia, a disease resulting from an obvious angiogenic imbalance, proteinuria and hypertension implicate the endothelium as the target of the disease[11]. We included proteinuria and hypertension as evaluations of endothelial injury to explore the factors involved in endothelial injury in IgAN. Moreover, the newly proposed Oxford-MEST histological classification for IgAN uses E scores to evaluate endocapillary proliferation. Therefore we used Oxford-E scores to evaluate the histological endothelial injury. Furthermore, vWF, a widely agreed biochemical marker for endothelial injury, was also used. We included proteinuria, hypertension, histological E scores and plasma vWF levels as parameters to evaluate endothelial injury in the present study.

#### Proteinuria

**Figure 2:**
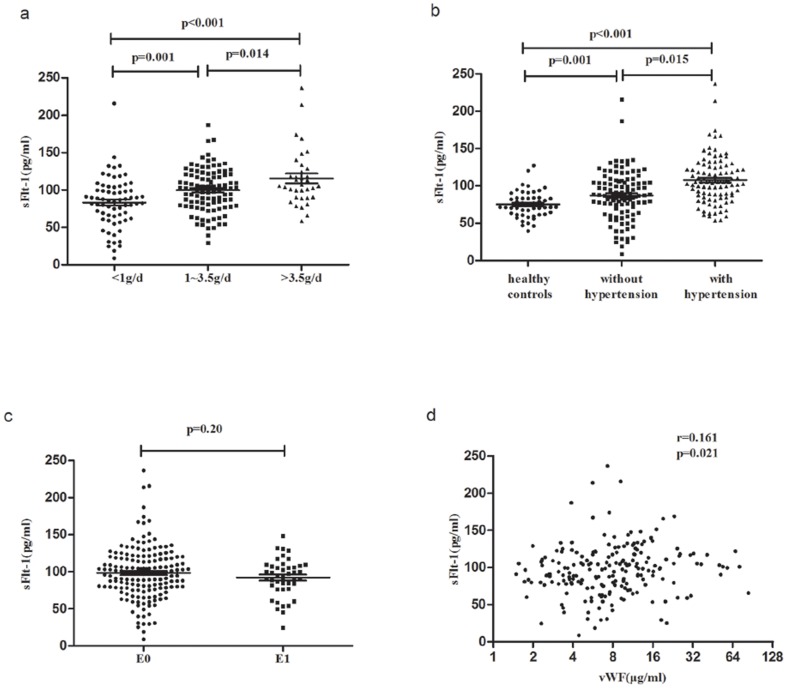
Correlations of sFlt-1 with proteinuria, hypertension, Oxford-E score and vWF levels. **a**. Scatter plot showing the distribution of sFlt-1 levels in IgAN patients according to different degrees of proteinuria. Patients with mild proteinuria (<1 g/d) showed lower plasma sFlt-1 compared to patients with moderate (1–3.5 g/d) and severe proteinuria (>3.5 g/d). **b**. Scatter plot showing the distribution of sFlt-1 levels of IgAN patients according to hypertension. IgAN patients with hypertension presented significantly higher sFlt-1 levels than patients without hypertension and healthy volunteers. Furthermore, IgAN patients without hypertension also showed higher sFlt-1 levels than healthy volunteers. **c**. Scatter plot showing the distribution of sFlt-1 levels in IgAN patients according to Oxford-E score. The sFlt-1 levels were similar in the E1 group compared to the E0 group. **d**. Scatter plot showing the correlation between sFlt-1 and vWF levels. Plasma sFlt-1 levels were significantly correlated with vWF levels.

A correlation was observed between plasma sFlt-1 and proteinuria in IgAN patients. Patients with mild proteinuria (<1 g/d) showed lower plasma sFlt-1 levels compared to patients with moderate (1–3.5 g/d) and severe proteinuria (>3.5 g/d) (severe *vs.* moderate *vs.* mild: 115.95±39.09 *vs.* 99.89±28.55 *vs.* 83.24±33.92 pg/ml, severe *vs.* mild: p<0.001, moderate *vs.* mild: p = 0.001, severe *vs.* moderate: p = 0.014) (Figure 2).

#### Hypertension

We found elevated plasma sFlt-1 levels in IgAN patients. IgAN patients with hypertension presented with significantly elevated sFlt-1 levels compared to patients without (107.87±31.94 *vs.* 87.32±32.76 pg/ml, p = 0.015) and healthy volunteers (107.87±31.94 *vs.* 75.06±17.55 pg/ml, p<0.001). Furthermore, IgAN patients without hypertension also showed higher sFlt-1 levels than healthy volunteers (107.87±31.94 *vs.* 75.06±17.55 pg/ml, p = 0.001) (Figure 2). These results indicated a correlation between plasma sFlt-1 and clinical hypertension in IgAN patients.

#### E scores of Oxford classification

No correlation was observed between Oxford-E scores and sFlt-1. IgAN patients with scores of E0 and E1 did not show significantly different plasma sFlt-1 levels (E1 *vs.* E0: 92.13±25.68 *vs.* 98.35±35.71 pg/ml, p = 0.20) (Figure 2).

#### Von Willebrand Factor

We first investigated the correlation between sFlt-1 and vWF. sFlt-1 was positively correlated with vWF in IgAN patients (r = 0.161, p = 0.021), which is a specific endothelial injury marker (Figure 2).

**Figure 3:**
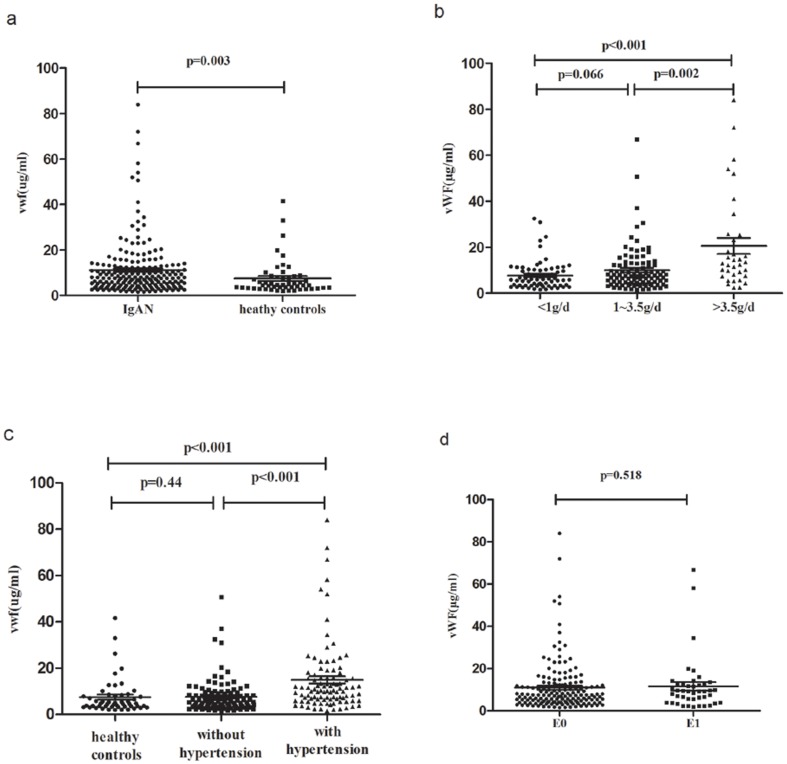
Correlations of vWF with proteinuria, hypertension and Oxford-E score. **a**. Scatter plot showing the distribution of vWF levels in IgAN patients and healthy volunteers. IgAN patients showed significantly higher vWF levels than healthy volunteers. **b**. Scatter plot showing the distribution of vWF levels of IgAN patients according to different degrees of proteinuria. Patients with mild proteinuria (<1 g/d) showed lower plasma sFlt-1 levels compared to patients with moderate (1–3.5 g/d) and severe proteinuria (>3.5 g/d). **c**. Scatter plot showing the distribution of vWF levels in IgAN patients with or without hypertension. IgAN patients with hypertension presented significantly higher vWF levels than patients without hypertension and healthy volunteers. However, IgAN patients without hypertension showed similar vWF levels compared to healthy volunteers. **d**. Scatter plot showing the distribution of sFlt-1 levels of IgAN patients according to Oxford-E score. The sFlt-1 levels were similar in the E1 group compared with the E0 group.

We next compared plasma vWF levels between patients and volunteers. IgAN patients showed significantly higher vWF levels than healthy volunteers (11.08±12.16 *vs.* 7.48±7.72 µg/ml, p = 0.003) (Figure 3), in accordance with sFlt-1. Furthermore, correlations between vWF and proteinuria, hypertension, and Oxford-E score were also analyzed. IgAN patients with mild proteinuria showed lower levels of vWF than patients with moderate proteinuria (mild *vs.* moderate: 7.78±6.34 *vs.* 10.01±9.58 µg/ml, p = 0.066) and severe proteinuria (mild *vs.* severe: 7.78±6.34 *vs.* 18.96±19.80 µg/ml, p<0.001). Patients with moderate proteinuria had significantly lower vWF levels than patients with severe proteinuria (10.01±9.58 *vs.* 18.96±19.80 µg/ml, p = 0.002) (Figure 3). Patients with hypertension showed significantly higher levels of vWF than patients without hypertension (14.92±15.0*7 vs.* 7.63±7.26 µg/ml, p<0.001) and healthy volunteers (14.92±15.07 *vs.* 7.48±7.72 µg/ml, p<0.001), but there was no difference between IgAN patients without hypertension and healthy volunteers (7.63±7.26 *vs.* 7.48±7.72 µg/ml, p = 0.44) (Figure 3). IgAN patients with scores of E0 and E1 did not show significantly different plasma vWF levels (E1 *vs.* E0: 11.61±12.90 *vs.* 10.94±11.99 pg/ml, p = 0.518) (Figure 3).

Our results indicated that elevated sFlt-1 levels in IgAN patients were significantly correlated with the well-proven endothelial injury marker, vWF.

### Plasma vWF levels, but not sFlt-1 levels, correlated with eGFR

**Figure 4:**
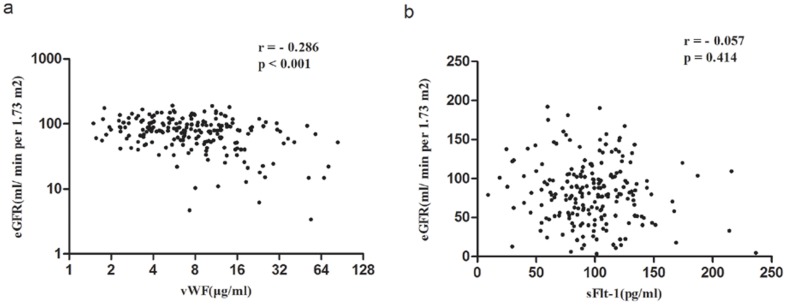
Correlation of vWF and sFlt-1 levels with eGFR. **a**. Plasma vWF levels were negatively correlated with eGFR. **b**. Plasma sFlt-1 levels showed no correlation with eGFR.

Renal excretion greatly influences many circulating elements. Therefore, we further investigated the correlation of elevated sFlt-1 and vWF with eGFR in patients with IgAN, whose renal excretion function is often impaired. Our data showed that plasma vWF levels were negatively correlated with eGFR in IgAN patients (r = −0.286, p<0.001; Figure 4), but no correlation between sFlt-1 and eGFR was observed (r = −0.057, p = 0.414; Figure 4).

## Discussion

IgA nephropathy is a common, complex disease with variable manifestations. Endothelial dysfunction, which is observed in a considerable proportion of patients with IgAN, is one of these manifestations, but the mechanism that leads to this dysfunction is not clear. The present study explored the underlying mechanism of endothelial injury in IgAN patients and identified an angiogenic imbalance of VEGF/sFlt-1 in IgAN patients for the first time.

VEGF is a potent mitogen for endothelial cells, and it plays a central role in maintaining the integrity of the endothelial lining. sFlt-1, also known as soluble VEGFR-1, acts as a VEGF antagonist. The ratio of VEGF/sFlt-1 is often used to evaluate the balance of angiogenesis. It is very interesting that both sFlt-1 and VEGF/sFlt-1, but not VEGF, were significantly different between IgAN patients and healthy volunteers in the present study. These results implied that elevated sFlt-1 may be the most important contributor to the angiogenic imbalance in IgAN, which is different from diabetic nephropathy and pre-eclampsia in which both a decreased VEGF and increased sFlt-1 have been reported[11], [22]. Elevated sFlt-1 in patients with IgAN was further confirmed in an independent replication cohort in our study. Another important thing to note is that, sFlt-1 levels are significantly elevated to over 3000 pg/ml in patients with pre-eclampsia. Even in normal-term pregnancy women, the sFlt-1 levels were already 10 times higher than healthy volunteers[11]. The placenta is the main source of elevated sFlt-1[23], although one recent study found that platelet-monocyte aggregate-derived sFlt-1 also contributed to the pathogenesis of pre-eclampsia [24]. The sFlt-1 levels in patients with IgAN were elevated slightly but significantly compared to pre-eclampsia. Further in depth studies are required to identify the source and the variance of elevated sFlt-1 in IgAN.

We further explored the correlations of elevated sFlt-1 with proteinuria, hypertension, Oxford-E scores and vWF levels (Figure S1). Previous studies in animal models and humans have proven the importance of VEGF signaling in maintaining the integrity of glomerular filtration barrier[25], [26]. A defective survival of glomerular endothelial cells would occur in the absence of VEGF signaling, which leads to proteinuria. Our study found that IgAN patients with severe proteinuria had higher sFlt-1, and IgAN patients with mild proteinuria had lower sFlt-1. These results imply the following underlying mechanism: increased sFlt-1, through its inhibition of VEGF, interfered with glomerular endothelial survival, and finally destroyed the glomerular filtration barrier to result in proteinuria.

Except for renal endothelial injury, VEGF signaling dysfunction can also lead to systemic endothelial injury. Maynard et al. reported that sFlt-1 administration to pregnant rats induced hypertension and glomerular endotheliosis[11].We found a correlation between increased sFlt-1 and hypertension in our population, which suggested that elevated sFlt-1 might be a factor of systemic endothelial cell injury.

A correlation between plasma sFlt-1 levels and Oxford-E scores was not observed in the present study. We grouped patients into groups with hypertension and without hypertension, and correlations were not found in either group (Figure S2). Similarly, vWF levels also failed to show correlations with Oxford-E scores. We have some considerations at this point. At first, the E score was a semi-quantitative pathological parameter, and although it worked well for the prediction of prognosis, it might not be sensitive enough to evaluate variations of endothelial injury in IgAN. Secondly, Oxford-E scores were evaluated mainly in the endocapillary hypercellularity, which may have limited power for these scores to reflect the sFlt-1-induced endothelial injury for decreased endothelial survival. Thirdly, it would be more reasonable to analyze the correlation of Oxford-E scores with local sFlt-1 levels rather than plasma sFlt-1 levels because there may be some difference.

vWF is a well-known marker for endothelial dysfunction. Our present study observed elevated vWF levels in patients with IgAN, which also correlated with clinical proteinuria and hypertension in IgAN and confirmed the existence of endothelial injury in IgAN. Moreover, sFlt-1 levels were positively correlated with vWF in our patients with IgAN, which provided direct evidence to suggest sFlt-1 as a causal factor for endothelial injury in IgAN.

Plasma vWF levels are widely used as a biomarker for endothelial injury in several conditions. We found a negative correlation of vWF levels with eGFR in our patients with IgAN. However, sFlt-1 levels seemed not influenced by eGFR. Therefore, sFlt-1 might be a more suitable biomarker for endothelial injury for patients with chronic kidney diseases, especially those patients with impaired renal function.

Notably, our present study was a cross-sectional study. We only detected the sFlt-1 levels on the day of renal biopsy for patients with IgAN. Therefore, we cannot currently evaluate the variability of sFlt-1 measurements over time. The identification of sFlt-1 as a correlated biomarker for endothelial injury in our study will be of great clinical importance for investigating sFlt-1 levels in a follow-up population of IgAN patients.

In conclusion, the present study found elevated sFlt-1 in IgAN patients for the first time and further identified its correlation with proteinuria, hypertension and vWF levels. Our results suggest elevated sFlt-1 as one of the contributors to endothelial injury in IgA nephropathy.

## Supporting Information

Figure S1 **Flow chart showing the process of the present study.** VEGF/sFlt-1, sFlt-1 and VEGF levels were firstly compared in discovery cohort between 96 patients with IgAN and 22 healthy volunteers. Next, the identified contributor (sFlt-1) was confirmed in a replication cohort (109 IgAN patients, 30 healthy volunteers). At last, the correlations of sFlt-1 with hypertension, proteinuria, Oxford-E score and plasma vWF, were evaluated in the combined 205 patients with IgAN.
(TIF)

Figure S2 **sFlt-1 levels in patients with E1 and E0 in hypertension group and without hypertension group.** In patients with IgAN, sFlt-1 levels were similar in those with Oxford-E1 and Oxford-E0, either in hypertension group (**a**) or without hypertension group (**b**).
(TIF)
