# Supplementary figures and images for: Elevated Soluble VEGF Receptor sFlt-1 Correlates with Endothelial Injury in IgA Nephropathy
Source: PLoS One. 2014 Jul 9;9(7):e101779. doi: 10.1371/journal.pone.0101779 (PMC4090210; doi:10.1371/journal.pone.0101779)

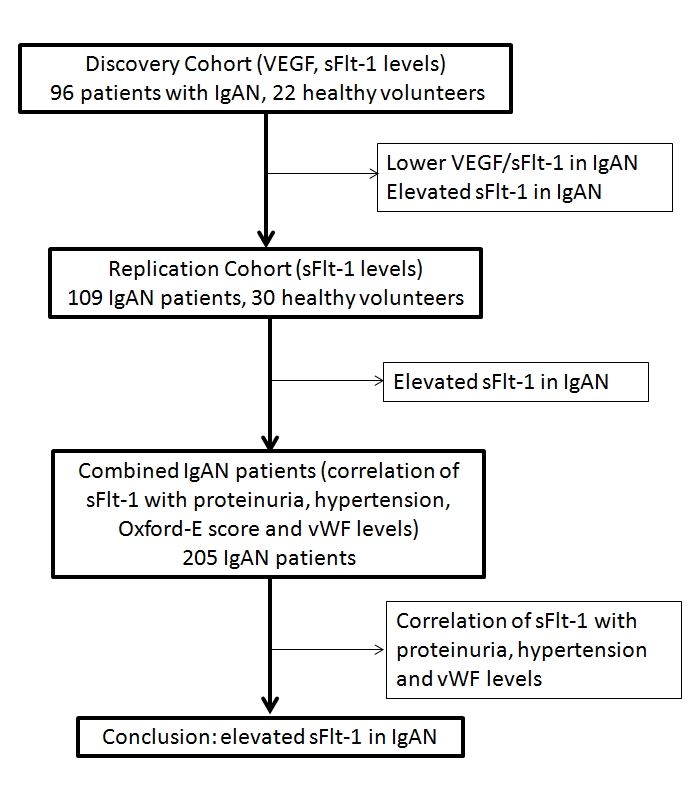

Supplement: Figure S1 — Flow chart showing the process of the present study. VEGF/sFlt-1, sFlt-1 and VEGF levels were firstly compared in discovery cohort between 96 patients with IgAN and 22 healthy volunteers. Next, the identified contributor (sFlt-1) was confirmed in a replication cohort (109 IgAN patients, 30 healthy volunteers). At last, the correlations of sFlt-1 with hypertension, proteinuria, Oxford-E score and plasma vWF, were evaluated in the combined 205 patients with IgAN. (TIF) [file pone.0101779.s001.tif]

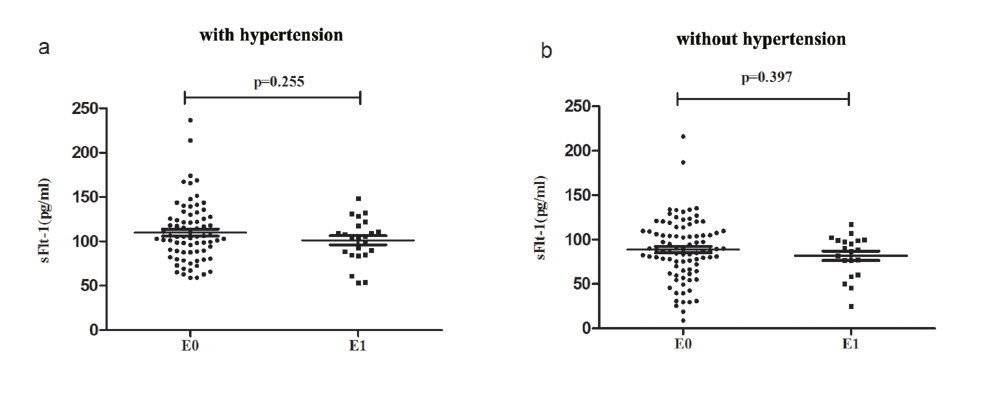

Supplement: Figure S2 — sFlt-1 levels in patients with E1 and E0 in hypertension group and without hypertension group. In patients with IgAN, sFlt-1 levels were similar in those with Oxford-E1 and Oxford-E0, either in hypertension group (a) or without hypertension group (b). (TIF) [file pone.0101779.s002.tif]
